# Effect of Influenza Vaccine on Prevention of Acute Attack of Chronic Airway Disease in Elderly Population

**DOI:** 10.3390/vaccines10101750

**Published:** 2022-10-19

**Authors:** Kun Gao, Guangbo Qu, Cuihong Zhang, Huaibiao Li, Liang Sun

**Affiliations:** 1 Department of Geriatrics, Fuyang People’s Hospital of Anhui Province, Fuyang 236006, China; 2 Department of Epidemiology and Health Statistics, Anhui Medical University, Meishan Road 81, Hefei 230032, China; 3 Fuyang Center for Disease Control and Prevention, Fuyang 236069, China

**Keywords:** influenza vaccine, chronic airway disease, acute attack, elderly population

## Abstract

This study investigated the effect of influenza vaccination on prevention of acute attacks in elderly patients with chronic airway disease and provides evidence for the prevention and control strategy of chronic airway disease in the elderly population. A total of 348 elderly patients in Linquan County, Anhui Province, China, who were also in stationary phases of chronic airway disease and were vaccinated with either the tetravalent or trivalent influenza vaccine were selected. The number of patients with acute attacks, the number of outpatients with acute attacks, the number of outpatients, the number of inpatients, the total cost of patients, the cost of outpatients, the cost of hospitalization, and the length of hospitalization were collected before vaccination and after a one-year follow-up. There was no significant difference in age and sex ratio among the two vaccination groups. The ratios of acute attacks, outpatient visits, and hospitalizations and number of outpatient visits, number of hospitalizations, total medical expenses, outpatient expenses, and hospitalization expenses were significantly higher before vaccination than those after vaccination in both the trivalent-vaccination group and tetravalent-vaccination group. Additionally, there was no significant difference in the length of stay between before and after vaccination in either the trivalent-vaccination group or tetravalent-vaccination group. The protection effect between the trivalent-vaccination group and tetravalent-vaccination group was not significant. Influenza vaccination can effectively prevent the acute attack of chronic airway disease and delay the progress of chronic airway disease.

## 1. Introduction

Chronic airway disease is a group of diseases, characterized by airflow limitation, dyspnea, and shortness of breath, mainly including chronic obstructive pulmonary disease (COPD), chronic bronchitis, emphysema, chronic bronchitis with emphysema, asthma, and bronchiectasis [1]. COPD is closely related to chronic bronchitis and emphysema, which can be diagnosed with continuous airflow limitation in patients with chronic bronchitis and emphysema [2]. According to the estimation of the World Health Organization (WHO) in 2016, about 339 million people in the world suffered from asthma [3], and about 251 million people suffered from COPD [4]. In 2016, COPD ranked third among the top ten causes of death in the world, resulting in three million deaths [5]. Hundreds of millions of people in the world suffer daily from the hazards and pain of chronic airway disease [6]. Chronic airway disease is also one of the main causes of death in China. It is reported that there are nearly 100 million COPD patients in China, and the prevalence of COPD is over 27% among patients over 60 years old and increases along with age. The mortality of COPD reaches 79.44 per 100,000, which is the third leading cause of death in China [7,8]. Therefore, early prevention and intervention of chronic airway disease are urgently needed, considering its high prevalence and mortality.

Recently, chronic airway disease has become an important public health issue worldwide. Intervention during the stationary phase of chronic airway disease may be an important way to reduce the incidence of acute exacerbation and improve lung function. Respiratory tract infection is one of the main causes of acute exacerbation of chronic airway disease [2]. Thus, influenza vaccination may be beneficial in preventing chronic airway disease, given its role in preventing respiratory infections. Actually, it has been reported that influenza vaccination can prevent acute exacerbation of COPD, which can not only reduce the mortality but also reduce the severity of the disease, play an important role in reducing symptoms, and improve the quality of life of patients with chronic airway disease [9,10]. Based on the evidence of randomized controlled trials, a review has summarized that inactivated influenza vaccine did decrease ‘flare ups’ of COPD, especially those associated with the influenza virus itself, but adding a live attenuated virus to the inactivated virus did not add any further protection for the participants [11]. In addition, a systematic review and meta-analysis identified a protective role of influenza vaccination in COPD patients and strongly recommended a yearly influenza vaccination for all COPD patients, especially those with severe airflow limitation [12]. However, evidence of the effect of influenza vaccine on the prevention of acute attack of chronic airway disease is limited. Furthermore, it should be noted that elderly people are susceptible to respiratory diseases and at higher risk of the acute attack of chronic airway disease. This study was therefore conducted to evaluate the effect of influenza vaccination on reducing the acute attacks of elderly patients with chronic airway disease, as well as providing evidence for the optimization of prevention and control strategies of chronic airway disease in China.

## 2. Methods

### 2.1. Participants

From information in the agricultural union report of Songji Town, Linquan County, Anhui Province, patients diagnosed with chronic airway disease in 2019 were selected. The inclusion criteria were ① age was 65 years old and above; ② patients without acute attack of chronic bronchitis, chronic bronchitis with emphysema, chronic obstructive pulmonary disease, etc.; ③ patients without allergic history, history of congenital, hereditary, or immunodeficiency diseases, history of nervous system diseases or mental diseases, and fever; ④ no influenza vaccine was received in the past year; ⑤ informed consent of subjects.

### 2.2. Exposures

The implementation time of the trail was from December 2019 to December 2020. According to the Technical Guidelines for Influenza Vaccination in China [13], trivalent and quadrivalent influenza vaccines are currently mainly used in China, and quadrivalent influenza vaccines can give more comprehensive protection because they can protect against more types of influenza viruses. The quadrivalent influenza vaccine contains antigen components of influenza A (H1N1), influenza A (H3N2), influenza B Yamagata strain, and influenza B Victoria strain. Compared with the trivalent influenza vaccine, the quadrivalent influenza vaccine contains an additional antigen of Yamagata lineage of influenza B virus, which can cover more influenza-virus circulating types. In this study, we mainly compared the effects of protection of the trivalent and quadrivalent influenza vaccines. Therefore, the included subjects were willing to be vaccinated with either the trivalent or tetravalent influenza vaccine.

### 2.3. Outcomes

The primary outcome was determining the ratio of acute attacks of chronic airway disease. The acute attacks of chronic airway disease were denied as being the acute worsening of respiratory symptoms, including the increase of cough, sputum production, and dyspnea, which requires a change in treatment strategy [14,15]. The secondary outcomes were the number of outpatients, the number of outpatient visits, the number of inpatients, the number of hospitalizations, the total medical cost of patients, outpatient expenses, hospitalization expenses, and the length of stay of patients with acute attacks. We compared the differences of these outcomes among patients before and after vaccination and between groups with different influenza vaccines.

### 2.4. Statistical Analysis

SPSS 22.0 and Excel software were used for statistical analysis. The counting data were described as number and rate, and continuous data were presented as mean and standard deviation (mean ± SD). Firstly, to compare the demographic characteristics between trivalent-vaccination and tetravalent-vaccination groups, chi-squared tests were performed. Secondly, the comparison of number of acute attacks, outpatient visits, and hospitalizations between before and after trivalent or quadrivalent vaccination was conducted using chi-squared tests. Thirdly, the comparison between before and after trivalent or quadrivalent vaccination in the number of outpatient visits, number of hospitalizations, total medical expenses, outpatient expenses, hospitalization expenses, and length of hospital stay were performed using the paired t-test. Finally, we also explored whether there is a difference in the impact of two kinds of vaccinations on medical and health costs; thus, independent t-tests were used for the comparison of the difference before and after vaccination in the number of outpatient visits, number of hospitalizations, outpatient expenses, hospitalization expenses, and length of hospital stay between trivalent-vaccination and tetravalent-vaccination groups. The statistically significant difference between groups was set at a *p*-value less than 0.05. Because the details of medical expenses could not be obtained before vaccination, the actual total amounts of medical-insurance reimbursement in that year were used for analysis.

## 3. Results

### 3.1. Description of Participants

A total of 348 subjects were included in this study, aged between 66 and 95 years old, with an average age of (74.70 ± 5.46) years old. The median age of the trivalent group was (74.89 ± 5.49) years old, and that of the tetravalent group was (74.22 ± 5.39) years old. There was no significant difference in age between the two groups (*t* = 1.038, *P* = 0.300). Among all subjects, 61.21% were male, 62.90% were male in the trivalent group, and 57.00% were male in the tetravalent group. There was no significant difference in gender between the two groups (*χ^2^* = 1.046, *P* = 0.306). During the follow-up, 14 patients died, and the main causes of death were COPD, lung cancer, and coronary heart disease. The details are presented in Table 1.

### 3.2. Comparison of Acute Exacerbation of Chronic Airway Disease in Trivalent-Influenza-Vaccination Group before and after Vaccination for One Year

**Figure 1:**
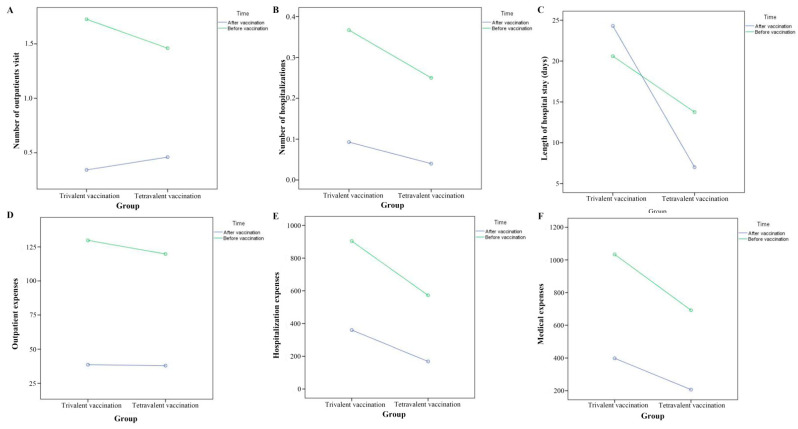
The variation in number of outpatient visits, the number of hospitalizations, the total medical cost of patients, outpatient expenses, hospitalization expenses, and the length of stay of patients before and after influenza vaccination among two groups. (**A**) number of outpatient visits; (**B**) number of hospitalizations; (**C**) length of hospital stay; (**D**) outpatient expenses; (**E**) hospitalization expenses; (**F**) total medical cost.

A total of 248 of patients were included in the trivalent-vaccination group. As presented in Table 2, the ratios of acute attacks, outpatient visits, and hospitalizations were significantly higher before vaccination than those after vaccination. In addition, the number of outpatient visits, number of hospitalizations, total medical expenses, outpatient expenses, and hospitalization expenses were significantly higher before vaccination than those after vaccination (Figure 1). Before vaccination, among the patients presenting with an acute attack, the minimum medical expense was RMB 16.64 (Chinese yuan), the maximum was RMB 16745.01, and the average medical expense was RMB (904.25 ± 2308.07). After vaccination, the minimum medical expense was RMB 42.90, the maximum was RMB 26793.81, and the average medical expense was RMB (359.79 ± 2196.30). The medical expenses were significantly higher before vaccination than those after vaccination (*P* = 0.003). The mean length of hospital stay was (13.46 ± 8.19) days before vaccination and (19.79 ± 35.01) days after vaccination. There was no significant difference in the length of hospital stay before and after vaccination (*P* = 0.770) (Figure 1).

### 3.3. Comparison of Acute Exacerbation of Chronic Airway Disease in Tetravalent-Influenza-Vaccination Group before and after Vaccination for One Year

A total of 100 subjects were enrolled in the tetravalent-vaccination group. The ratios of acute attacks, outpatient visits, and hospitalizations were significantly higher before vaccination than those after vaccination (Table 2 and Figure 1). In addition, the number of outpatient visits, number of hospitalizations, total medical expenses, outpatient expenses, and hospitalization expenses were also significantly higher before vaccination than those after vaccination (Table 2 and Figure 1). Before vaccination, among the patients with medical expenses, the minimum value was RMB 20.80, the maximum value was RMB 9394.86, and the average medical expense was RMB (692.01 ± 1809.90). After vaccination, among the patients with medical expenses, the minimum value was RMB 40, the maximum value was RMB 6935.51, and the average medical expense was RMB (206.57 ± 947.96). The medical expenses were significantly higher before vaccination than those after vaccination (*P* = 0.001). The mean length of hospital stay was (10.06 ± 6.01) days before vaccination and (7.00 ± 2.71) days after vaccination. There was no significant difference in the length of hospital stay before and after vaccination (*P* = 0.283).

### 3.4. Comparison of Protection Effect in Different Influenza-Vaccination Groups

No significant differences were found between the trivalent and tetravalent groups in the number of outpatient visits, number of hospitalizations, total medical expenses, outpatient expenses, hospitalization expenses, and length of hospital stay before and after vaccination (Table 2).

## 4. Discussion

Chronic airway disease seriously affects the quality of life of patients, and its high incidence rate, high disability rate, and high mortality bring heavy financial burden to patients and their families and society [7]. This study showed that the ratios of acute attacks, hospitalization times, treatment costs, and hospitalization times of patients with chronic airway disease who received the influenza vaccine were lower than those before vaccination, which is consistent with the results of other studies [10,16,17], which indicates that the influenza vaccination can prevent the acute attacks of chronic airway disease. Respiratory diseases are usually avoidable, and the cost of prevention is only a small part of treatment. The ability of the world to control and eliminate respiratory diseases depends on public health measures, including awareness-raising, education, and capacity-building [6]. Influenza vaccination is an important strategy to enhance the immunity of patients with chronic airway disease, and vaccination is the safest and most effective method. Therefore, the government and policymakers should include flu vaccines in healthcare strategies or free vaccination strategies for elderly people.

The beneficial effect of influenza vaccination on patients with chronic airway disease may be attributed to several reasons. Firstly, many factors can influence the exacerbation of chronic airway disease, including viral infections, bacterial infections, air pollution, temperature [18,19], and acute exacerbations caused by viruses, which tend to be more severe, last longer, and have a longer hospital stay [20]. Influenza vaccination is known to be effective in preventing influenza-related acute respiratory disease, which can avoid the subsequent adverse developments caused by acute exacerbation in patients. Secondly, it should be noted that the host’s immunity to bacteria, co-infection, and secondary bacterial infection can be decreased by influenza vaccination [21,22]. Bacterial infection, secondary to viral infection, increases the risk of acute exacerbation and mortality of chronic airway disease [23,24]. Therefore, the incidence of acute exacerbation or severe pneumonia can be reduced by the influenza vaccination for its role in prevention of the occurrence of influenza and reducing the incidence of viral and bacterial co-infection of the upper and lower respiratory tract in patients. Thirdly, it has been demonstrated that influenza vaccination can also reduce flu-related acute-lower-respiratory infections in the elderly population and hospitalized patients [25,26]. Severe airflow limitation due to lower respiratory tract infection is also a risk factor for the exacerbation of chronic airway disease [27,28]. All these causes suggest a strong recommendation for influenza vaccination in elderly patients with chronic airway disease.

This study found that there was no significant difference in medical expenses for patients with acute exacerbations before vaccination compared with those after vaccination, and the difference of length of hospital stay before and after vaccination was also not statistically significant, which may be caused by the same treatment methods, issued examinations, and drugs of the subjects with acute attacks of chronic airway disease. We also found that the length of stay of hospitalized patients is more than seven days, which may be related to the local habit of taking seven days as a hospitalization cycle. Therefore, this study was limited by hospitalization cycle, and, as a consequence, we were unable to judge whether influenza vaccination could reduce the severity of the acute attack of chronic airway disease by treatment cost and hospital stay.

There is no difference between trivalent influenza vaccine and tetravalent influenza vaccine in preventing the acute attack of chronic airway disease or reducing hospitalization times, treatment costs, and length of hospital stay. These findings indicate that different influenza vaccines may have the same effect in preventing the acute attack of chronic airway disease. However, this should be extrapolated carefully because the main epidemic strains of influenza virus in the northern hemisphere in 2020 are H3N2, B Victoria, and H1N1 [29]. Trivalent vaccines include the main epidemic strains in the northern hemisphere in 2020, but if trivalent vaccines do not include the main epidemic strains in the future and tetravalent vaccines include the main epidemic strains, the effect of different influenza vaccines on preventing the acute attack of chronic airway disease may be different. The effect of different influenza vaccines on preventing the acute exacerbation of chronic airway disease needs further long-term research to verify.

Several limitations should be noted. First, in 2020, novel coronavirus pneumonia affected the number of patients with chronic airway disease in the hospital. Second, affected by the novel coronavirus pneumonia, masks are used to reduce the chance of infection, which resulted in fewer instances of chronic airway disease and overestimated the effect of influenza vaccine in preventing the acute attack of chronic airway disease. Finally, we only analyzed some indicators of elderly patients with chronic airway disease who were vaccinated with the influenza vaccine within one year. The improvement of symptoms of elderly patients with chronic airway disease after long-term vaccination once a year still needs further follow-up observation.

## 5. Conclusions

Influenza vaccination can reduce the number of outpatient visits, hospitalization times, total treatment costs, outpatient costs, hospitalization costs, and hospitalization days for patients with chronic airway disease. It seems that the prevention effects of the trivalent influenza vaccine and tetravalent influenza are equivalent. The elderly patients with chronic airway diseases can be vaccinated with the influenza vaccine to prevent the acute attack of chronic airway disease and delay the progress of the disease.

## Figures and Tables

**Table 1:** Characteristics of all subjects.

Characteristics	All Subjects (*n* = 348)	Trivalent-Vaccination Group (*n* = 248)	Tetravalent-Vaccination Group (*n* = 100)	*t/* *χ* ^2^	*P*-Value
Age (years, mean ± SD)	74.70 ± 5.46	74.89 ± 5.49	74.22 ± 5.39	1.038	0.300
Age group (*n*, %)					
66~70	92 (26.44)	64 (25.80)	28 (28.00)	2.019	0.732
71~75	104 (29.89)	71 (28.63)	33 (33.00)		
76~80	98 (28.16)	71 (28.63)	27 (27.00)		
81~85	47 (13.50)	36 (14.52)	11 (11.00)		
≥86	7 (2.01)	6 (2.42)	1 (1.00)		
Sex (*n*, %)					
Male	213 (61.21)	156 (62.90)	57 (57.00)	1.046	0.306
Female	135 (38.79)	92 (37.10)	43 (43.00)		
Survival state					
Death	14 (4.02)	12 (4.84)	2 (2.00)	0.843	0.359
Survival	334 (95.98)	236 (95.16)	98 (98.00)		
Cause of death					
COPD	6 (42.86)	6 (50.00)	0 (0.00)	1.241	0.265
Lung cancer	3 (21.43)	2 (16.67)	1 (50.00)	NA	1.000
CHD	2 (14.29)	2 (16.67)	0 (0.00)	NA	1.000
Gastric cancer	1 (7.14)	0 (0.00)	1 (50.00)	NA	0.287
MI	1 (7.14)	1 (8.33)	0 (0.00)	NA	1.000
Suicide	1 (7.14)	1 (8.33)	0 (0.00)	NA	1.000

Abbreviations: COPD, chronic obstructive pulmonary disease; CHD, coronary heart disease; MI, myocardial infarction; NA: no value is available.

**Table 2:** Comparison of the medical situations of patients with chronic airway disease before and after influenza vaccination in one year.

Variables	Trivalent-Vaccination Group (*n* = 248)					Tetravalent-Vaccination Group (*n* = 100)					Comparison of the Difference between Groups	
	After Vaccination	Before Vaccination	t/*χ*^2^	*P*-Value	Difference	After Vaccination	Before Vaccination	Z/*χ*^2^	*P*-Value	Difference	*t/* *χ* ^2^	*P*-Value
Acute attack * (*n*, %)												
Yes	37 (14.92)	136 (54.84)	87.00	<0.001	NA	12 (12.00)	32 (32.00)	11.66	0.001	NA	NA	NA
No	211 (85.08)	112 (45.16)			NA	88 (88.00)	68 (68.00)			NA	NA	NA
Status of accessing medical service												
Outpatient visits (*n*, %)												
Yes	28 (11.29)	112 (45.16)	70.22	<0.001	NA	10 (10.00)	22 (22.00)	5.36	0.021	NA	NA	NA
No	220 (88.71)	136 (54.84)			NA	90 (90.00)	78 (78.00)			NA	NA	NA
Number of outpatient visits (mean ± SD)	0.34 ± 1.29	1.73 ± 3.81	−7.01	<0.001	−1.38 ± 3.11	0.46 ± 2.24	1.46 ± 4.67	−3.53	0.001	−1.00 ± 2.83	−1.067	0.287
Hospitalization (*n*, %)												
Yes	15(6.05)	59(23.79)	30.75	<0.001	NA	4 (4.00)	18 (18.00)	10.01	0.002	NA	NA	NA
No	233(93.95)	189(76.21)				96 (96.00)	82 (82.00)					
Number of hospitalizations (mean ± SD)	0.09 ± 0.42	0.37 ± 0.79	−6.06	<0.001	−0.27 ± 0.71	0.04 ± 0.20	0.25 ± 0.63	−3.91	<0.001	−0.21 ± 0.54	−0.914	0.362
Medical expenses ^#^ (mean ± SD)	398.41 ± 2219.30	1034.03 ± 2430.27	−3.47	0.001	−635.61 ± 2887.96	206.57 ± 947.96	692.01 ± 1809.90	−3.31	0.001	−485.44 ± 1468.22	−0.495	0.621
Outpatient expenses (mean ± SD)	38.63 ± 160.40	129.77 ± 291.80	−5.05	<0.001	−91.15 ± 284.42	37.94 ± 200.78	119.76 ± 487.07	−2.65	0.009	−81.82 ± 308.54	−0.270	0.787
Hospitalization expenses (mean ± SD)	359.79 ± 2196.30	904.25 ± 2308.07	−2.96	0.003	−544.46 ± 2892.95	168.63 ± 945.46	572.25 ± 1598.37	−2.96	0.004	−403.62 ± 1361.93	−0.466	0.641
Length of hospital stay (mean ± SD)	19.79 ± 35.01	13.46 ± 8.19	0.30	0.770	3.70 ± 38.91	7.00 ± 2.71	10.06 ± 6.01	−1.31	0.283	−6.75 ± 10.34	0.518	0.614

*: Represents patients who had acute attacks as long as there were records in the outpatient or inpatient department. ^#^: Represents the sum of outpatient and hospitalization expenses.

## Data Availability

Data are available under reasonable request from the corresponding author.
